# Supplementary material for: Reminders of Japanese redress increase Asian American support for Black reparations
Source: Commun Psychol. 2023 Nov 7;1:33. doi: 10.1038/s44271-023-00033-w (PMC11332238; doi:10.1038/s44271-023-00033-w)
Supplement: Supplementary file 1 — Supplementary information [file 44271_2023_33_MOESM1_ESM.pdf]

Supplementary Information

Reminders of Japanese redress increase Asian American support for Black reparations

Michael W. Kraus[1,2,3,*]

A. Chyei Vinluan[1]

[1]Yale University, School of Management, New Haven, CT, USA

[2]Yale University, Department of Psychology, New Haven, CT, USA

[3]Northwestern University, Department of Psychology, Evanston, IL, USA

[*]Corresponding Author Email: michael.kraus@northwestern.edu

Supplementary Methods

We used linguistic inquiry and word count (LIWC) software to determine if the manipulation was similar on the dimensions of affect, authority, and length (Tausczik & Pennebaker, 2010). Our analysis confirmed this expectation, in that no differences in affect, clout— a LIWC dictionary for words associated with authority and knowledge— and video length did not differ between control and intervention. The intervention video was wordier and contained bigger words, but we did not expect these differences to shape participant attitudes on Black reparations (see Table S1).

Table S1. A content comparison of the intervention and control videos using the linguistic inquiry and word count (LIWC) analyses for affect, word length, clout, word count, and number of video seconds. Chi-square equivalence tests reveal some similarities between the control and intervention videos on affect, apparent authority of speaker (clout), and duration.

LIWC Category	Reparations	Control	Chi-Square Equivalence Test	P Value
Affect	3.62	3.66	0.022	0.882
Big words	34.54	15.24	748.27	< .001
Clout	55.75	54.61	1.178	0.278
Word count	304	164	41.88	< .001
Seconds	143	136	0.176	0.675

Supplementary Note 1

We explored whether what participants wrote about during the intervention gave us clues about what they learned from the intervention and control conditions, and whether what they learned was related to support for Black reparations. We conducted this analysis by exploring

qualitative and quantitative analysis of text written by participants during exposure to the intervention (e.g., Callaghan et al., 2021). Both analyses reveal that the intervention condition in particular, increased writing about redress.

For the qualitative analysis, words related to justice showed up in 19 separate written responses in the control condition and 23 times in the intervention, but the tone and content of those justice statements differed considerably between the conditions. In the control condition, participants tended to write statements reflecting on the degree of injustice (e.g., "This is injustice to Japanese Americans," "it is not fair that Japanese Americans were discriminated and incarcerated just because of their ethnicity," and "I couldn't believe that the US would allow this injustice to fall upon the innocent US citizens"). However, zero of these statements took additional steps to suggest or recall corrections to this harm, in reparative economic action or otherwise.

In contrast, though justice reflections in the intervention condition also maligned the injustice of incarceration (e.g., "What happened to Japanese Americans was unjust and horrible"). Many of these written statements also tended to focus on gaining justice back through redress (e.g., "it was justified to seek reparations," "It was an awful thing they did to Japanese American citizens. I'm glad they got some justice," and "it was good they got some reparations in the end, something most other groups don't get"). Related to this point about gaining justice, 7 participants, all in the intervention condition spontaneously mentioned support for reparations for Black Americans in their written responses (e.g., "Black Americans also need reparations" and "why weren't their reparations similar to this for Black people and Native Americans?"). No mentions of Black Americans occur in the text responses of participants in the control condition.

For our quantitative analysis, we used the acquire dictionary of LIWC (Boyd et al., 2022) to explore whether increased writing related to gaining or getting justice through redress reflected in participant written text might be more prevalent in the intervention than the control condition, and

might explain the tendency for those in the intervention condition to support reparations for Black Americans. Our analysis found that words related to this state of acquiring (e.g., getting, gaining, got, take, taking) were found more in the intervention ($M = 0.77$, $SD = 2.17$) than the control condition ($M = 0.09$, $SD = 0.61$), $t(498) = 4.823$, $p < .001$. Moreover, uses of acquire related words were associated with support for reparations as measured by our composite reparations support index $r(498) = .125$, $p = .005$.

We used Process model 4 to conduct a mediation analysis with reparations support as the dependent variable, intervention as the independent variable, and acquire language coded by LIWC as the mediator. We used data from Study 2, and in the model we controlled for LIWC coded responses related to the amount of big words, positive and negative tone, and moral words. In addition, we controlled for demographic variables related to education and gender. The latter variables were added to the model because gender was associated with reparations support in Study 2, and education is a confound of writing proficiency.

The model found a significant effect of the intervention on the acquire language mediator $B = .633$ (.146), $t(492) = 4.351$, $p < .001$, and a significant effect of the acquire language mediator on reparations support $B = .0488$ (.022), $t(491) = 2.238$, $p = .0257$, as well as a significant effect of the intervention in the same model $B = .254$ (.072), $t(491) = 3.541$, $p < .001$. Bootstrapping analysis with 5,000 resamples revealed a significant indirect effect of the intervention on reparations support through the acquire language mediator $B = .031$(.011) *CI95%* [.012 to .054]. In the model, none of the control variables were related to using acquire language or to reparations save for gender $B = -.293$ (.071), $t(491) = -4.120$, $p < .001$.

Supplementary Note 2

In Study 1, $ts < 1.60$, p's $> .110$ and Study 2, t's < 1.02, p's > 0.31 we found no credible evidence that the intervention shifted participant attitudes with respect to any of our political

attitudes questions save for one case in Study 2, where Asian American participants reported that they faced fewer barriers in the intervention condition ($M = 3.59$) than in the control condition ($M = 3.29$), $t\,(498) = 2.239$, $p = .026$. We also explored moderation of the intervention effects by political attitudes related to conservatism, common fate judgments, and internalized model minority stereotypes as well as by demographic characteristics that included network diversity, immigrant generational status, and whether participants were Japanese American or not. For each analysis we control for age, educational attainment, and income given intercorrelations with these variables and political attitudes. All analyses are reported aggregated across the two studies.

Conservatism did not interact with the intervention to predict support for reparations, β = -.04, $t(808) = -1.314$, $p = .189$. In the model both conservatism , β = -.415, $t(808) = -13.116$, $p < .001$ and the intervention β = .147, $t(808) = 4.772$, $p < .001$ predicted support for reparations.

Common fate with Black Americans did not interact with the intervention to predict support for reparations, β = -.046, $t(808) = -1.481$, $p = .139$. In the model both common fate beliefs, β = .383, $t(808) = 12.180$, $p < .001$ and the intervention β = .158, $t(808) = 5.075$, $p < .001$ predicted support for reparations.

Beliefs that Asian Americans are harder working than other minority groups did not interact with the intervention to predict support for reparations, β = .014, $t(808) = 0.431$, $p = .666$. In the model both work ethic based stereotypes, β = -.287, $t(808) = -8.689$, $p < .001$ and the intervention β = .162, $t(808) = 4.997$, $p < .001$ predicted support for reparations.

Beliefs that Asian Americans do not experience the same discrimination as other minority groups did not interact with the intervention to predict support for reparations, β = .038, $t(808) = 1.127$, $p = .260$. In the model discrimination based model minority beliefs, β = .042, $t(808) = 1.230$, $p = .219$ did not predict reparations support but the intervention did β = .162, $t(808) = 4.808$, $p < .001$.

Network diversity did not interact with the intervention to predict support for reparations, β = .014, $t(808) = 0.415$, $p = .679$. In the model network diversity, β = -.065, $t(808) = -1.890$, $p = .058$ did not predict reparations support but the intervention did β = .162, $t(808) = 4.809$, $p < .001$.

Though immigrant generation did not significantly interact with the intervention to predict support for reparations that patterned of responses was indicative a potential relationship β = -.066, $t(808) = -1.940$, $p = .053$. In the model generation, β = -.054, $t(808) = -1.599$, $p = .110$ did not predict reparations support but the intervention did β = .163, $t(808) = 4.830$, $p < .001$. The interaction, though not significant, suggests that recent immigrant generations may have less knowledge of Japanese redress in particular, and so they were more influenced by the intervention than later generation immigrants.

The intervention did not depend on whether participants were Japanese American or not, β = .018, $t(808) = 0.276$, $p = .783$. In the model being Japanese American, β = .025, $t(808) = 0.718$, $p = .473$ did not predict reparations support but the intervention did β = .178, $t(808) = 2.782$, $p = .007$.

Supplementary Note 3

To examine the success of our random assignment manipulation, we examined between group differences in demographic variables (i.e., education, income, gender, age, Japanese ancestry) that should not change as a function of our experimental manipulation. The result of this preliminary analysis in Study 1 indicates the success of our manipulation—there was no statistically significant evidence for between condition differences in any of these demographic variables $ts < 0.962$, $ps > .337$. The same analysis yielded similar results in Study 2—there was no statistically significant evidence for between condition differences in any of these demographic variables $ts < 1.051$, $ps > .147$.

Supplementary Note 4

We pooled the analysis because the intervention condition and measures were identical across both studies, as were the observed effect sizes of the intervention on reparations support. Moreover, exploratory comparisons of correlations using a Fisher r to z transformation with a Bonferroni correction ($\alpha = .005$) for multiple comparisons found no credible evidence for differences between conditions in correlations between support for reparations and gender, income, education, model minority beliefs, feelings toward Black or Asian Americans, common fate beliefs, or network diversity between the samples $zs < 2.26$, $ps > .0238$. One difference emerged in associations between conservatism between the two samples $z = 3.24$, $p = .0021$, with the Study 1 sample reporting higher mean conservatism, but lower associations between conservatism and support for reparations.

Supplementary References

1. Tausczik, Y. R., & Pennebaker, J. W. (2010). The psychological meaning of words: LIWC and computerized text analysis methods. *Journal of language and social psychology*, *29*(1), 24-54.
2. Callaghan, B., Harouni, L., Dupree, C. H., Kraus, M. W., & Richeson, J. A. (2021). Testing the efficacy of three informational interventions for reducing misperceptions of the Black–White wealth gap. *Proceedings of the National Academy of Sciences*, *118*(38), e2108875118.
3. Boyd, R. L., Ashokkumar, A., Seraj, S., & Pennebaker, J. W. (2022). The development and psychometric properties of LIWC-22. *Austin, TX: University of Texas at Austin*, 1-47.
