# Supplementary material for: Reminders of Japanese redress increase Asian American support for Black reparations
Source: Commun Psychol. 2023 Nov 7;1:33. doi: 10.1038/s44271-023-00033-w (PMC11332238; doi:10.1038/s44271-023-00033-w)
Supplement: Supplementary file 3 — Reporting summary [file 44271_2023_33_MOESM3_ESM.pdf]

nature portfolio

Corresponding author(s): Michael Kraus

Last updated by author(s): Sep 29, 2023

Reporting Summary

Nature Portfolio wishes to improve the reproducibility of the work that we publish. This form provides structure for consistency and transparency in reporting. For further information on Nature Portfolio policies, see our Editorial Policies and the Editorial Policy Checklist.

Statistics

For all statistical analyses, confirm that the following items are present in the figure legend, table legend, main text, or Methods section.

n/a	Confirmed	
☐	☒	The exact sample size (*n*) for each experimental group/condition, given as a discrete number and unit of measurement
☒	☐	A statement on whether measurements were taken from distinct samples or whether the same sample was measured repeatedly
☐	☒	The statistical test(s) used AND whether they are one- or two-sided<br>*Only common tests should be described solely by name; describe more complex techniques in the Methods section.*
☐	☒	A description of all covariates tested
☐	☒	A description of any assumptions or corrections, such as tests of normality and adjustment for multiple comparisons
☐	☒	A full description of the statistical parameters including central tendency (e.g. means) or other basic estimates (e.g. regression coefficient) AND variation (e.g. standard deviation) or associated estimates of uncertainty (e.g. confidence intervals)
☐	☒	For null hypothesis testing, the test statistic (e.g. *F*, *t*, *r*) with confidence intervals, effect sizes, degrees of freedom and *P* value noted<br>*Give P values as exact values whenever suitable.*
☒	☐	For Bayesian analysis, information on the choice of priors and Markov chain Monte Carlo settings
☒	☐	For hierarchical and complex designs, identification of the appropriate level for tests and full reporting of outcomes
☐	☒	Estimates of effect sizes (e.g. Cohen's *d*, Pearson's *r*), indicating how they were calculated

Our web collection on statistics for biologists contains articles on many of the points above.

Software and code

Policy information about availability of computer code

Data collection	N/A
Data analysis	N/A

For manuscripts utilizing custom algorithms or software that are central to the research but not yet described in published literature, software must be made available to editors and reviewers. We strongly encourage code deposition in a community repository (e.g. GitHub). See the Nature Portfolio guidelines for submitting code & software for further information.

Data

Policy information about availability of data

All manuscripts must include a data availability statement. This statement should provide the following information, where applicable:

- Accession codes, unique identifiers, or web links for publicly available datasets
- A description of any restrictions on data availability
- For clinical datasets or third party data, please ensure that the statement adheres to our policy

Materials and data for the experiment and preregistration are available at the open science framework (data and materials: https://osf.io/mupkc/?view_only=926eca86d0ab4ec48c21fda51c62bf43; preregistration for study 1: https://osf.io/qxmak/?view_only=7790365ea0914406bf8b57301d4fb9e9); preregistration for study 2: https://osf.io/nb5zr/?view_only=0bba4852bab94af0afd77e789f796ce9).

Research involving human participants, their data, or biological material

Policy information about studies with human participants or human data. See also policy information about sex, gender (identity/presentation), and sexual orientation and race, ethnicity and racism.

Reporting on sex and gender	In Study 1, participants mostly self-identified as women (163), with 153 men, and 1 person identifying with another gender identity. In Study 2, Participants were mostly self-identified as men (307), with 186 women, and 4 people identifying with another gender identity.
Reporting on race, ethnicity, or other socially relevant groupings	In Study 1, we collected a sample of 329 Asian Americans from a variety of ethnic origin subgroups and generations in the US for an online study through Centiment Survey Panels, which recruits participants though online social networks for brief academic and marketing surveys. Respondents were paid $5 for a 10-minute survey and all participants consented to participate in the study which was approved by the institutional review board at Yale University. Asian subgroups included Chinese (91), Indian (55), Filipino (54), Japanese (30), Korean (24), Vietnamese (23), Taiwanese (10), and the remaining participants indicated membership in one of the remaining 19 Asian subgroups (i.e., Bhutanese, Bangladeshi, Burmese, Cambodian, Fijian, Hmong, Hong Konger, Indonesian, Malaysian, Mongolian, Native Hawaiian, Nepali, Pakistani, Samoan, Singaporean, Sri Lankan, Taiwanese, Thai, Tongan). In terms of U.S. immigration generational status, most of our sample was first (166) or second (113) generation followed by third (28), fourth (11), or fifth (3) generation.<br><br>In Study 2, we collected a sample of 500 Asian Americans from a variety of ethnic origin subgroups and generations in the US for an online study through Prolific Academic, a sign-up based online crowdsourced survey response platform with a reputation for high data quality.25,26 We used this alternative online sample to examine if our findings may generalize to this new group of Asian American participants. Respondents were paid $3 for a 10-minute survey and all participants consented to participate in the study which was approved by the institutional review board at Yale University. Asian subgroups included Chinese (146), Indian (61), Filipino (55), Korean (56), Vietnamese (76), Japanese (30), Taiwanese (26), and the remaining participants indicated membership in one of the remaining 19 subgroups. In terms of American generational status, most of our sample was first (138) or second (326) generation followed by third (12), fourth (18), or fifth (6) generation.
Population characteristics	see above
Recruitment	online data collection using crowdsourced panels from Centiment and Prolific services. These are advertised panels where people sign up to answer questions for marketing and academic research. People self-select into these panels and responses are collected in English, thus limiting their generalizeability.
Ethics oversight	Yale University institutional review board

Note that full information on the approval of the study protocol must also be provided in the manuscript.

Field-specific reporting

Please select the one below that is the best fit for your research. If you are not sure, read the appropriate sections before making your selection.

☐ Life sciences ☒ Behavioural & social sciences ☐ Ecological, evolutionary & environmental sciences

For a reference copy of the document with all sections, see nature.com/documents/nr-reporting-summary-flat.pdf

Behavioural & social sciences study design

All studies must disclose on these points even when the disclosure is negative.

Study description	Quantitative, experimental
Research sample	2 experiments with ~800 Asian American participants recruited from online crowdsourced panels.
Sampling strategy	Convenience samples from two different services (Centiment and Prolific). Self-selected but different in terms of age and politics to assess generalizeability of effects across samples.
Data collection	Qualtrics survey systems were used to collect data for the study.
Timing	June 30th, 2022 (Study 1) to August 7th<br>May 11th, 2023 (Study 2) to May 13th
Data exclusions	No data exclusions were made.
Non-participation	No participants dropped out once study began.
Randomization	Randomization occurred, analysis suggests randomization was successful for other demographic characteristics.

Reporting for specific materials, systems and methods

We require information from authors about some types of materials, experimental systems and methods used in many studies. Here, indicate whether each material, system or method listed is relevant to your study. If you are not sure if a list item applies to your research, read the appropriate section before selecting a response.

Materials & experimental systems

n/a	Involved in the study
☒	☐ Antibodies
☒	☐ Eukaryotic cell lines
☒	☐ Palaeontology and archaeology
☒	☐ Animals and other organisms
☒	☐ Clinical data
☒	☐ Dual use research of concern
☒	☐ Plants

Methods

n/a	Involved in the study
☒	☐ ChIP-seq
☒	☐ Flow cytometry
☒	☐ MRI-based neuroimaging
